# Management of Acute Promyelocytic Leukemia in the Elderly

**DOI:** 10.4084/MJHID.2013.045

**Published:** 2013-06-08

**Authors:** Francesco Lo-Coco, Roberto Latagliata, Massimo Breccia

**Affiliations:** 1 Department of Biomedicine and Prevention, University Tor Vergata, Rome, Italy; 2 Laboratory of Neuro-Oncohematology, Santa Lucia Foundation, Rome, Italy; 3 Department of Cellular Biotechnologies and Hematology, Sapienza University, Rome, Italy

## Abstract

Unlike other forms of AML, APL is less frequently diagnosed in the elderly and has a relatively favourable outcome. Elderly patients with APL seem at least as responsive to therapy as do younger patients, but rates of response and survival are lower in this age setting owing to a higher incidence of early deaths and deaths in remission when conventional treatment with ATRA and chemotherapy is used. Elderly APL patients are more likely to present with low-risk features compared with younger patients, and this may explain the relative low risk of relapse reported in several clinical studies. Alternative approaches, such as arsenic trioxide and gentuzumab ozogamicin have been tested with success in this setting and could replace in the near future frontline conventional chemotherapy and ATRA.

## Introduction

Acute promyelocytic leukemia (APL) is a peculiar subtype of acute myeloid leukemia (AML) associated with unique biologic features and requiring specific management. APL has become a well-recognized entity, by the French-American-British (FAB) identified as the M3 subtype, and by WHO classification, accounting for approximately 10% of cases of all AMLs.^1^,^2^,^3^ Presenting features include a severe coagulopathy due to excessive fibrinolysis and a frequently low white blood cell (WBC) count, although 20% to 30% patients present with WBC counts higher than 10,000/μL; striking sensitivity to anthracycline-containing chemotherapy and finally a unique responsiveness to differentiation treatment with retinoids. 4,5

A specific reciprocal translocation involving the long arms of chromosomes 15 and 17 is the marker of the disease.^6^ This chromosomal rearrangement involves the retinoic acid receptor alpha (RARα) gene on the long arm of chromosome 17 and the promyelocytic leukemia (PML) gene on chromosome 15. Two fusion genes are generated as a consequence, i.e. PML/RARα and the reciprocal RARα/PML.^7^, ^8^, ^9^ The median age at presentation is usually 40–45 years as reported in various clinical studies 10–14, whereas the incidence reported in patients aged from 60 to 70 years varies from 15 to 20% and for patients > 70 years from 1 to 6%. A population-based USA study reported an early death rate of 24% in patients aged 55 years or more^15^, while in a Swedish Leukemia Registry study the early death rate was 29% in all age groups and 50% in patients aged over 60 years.^16^ These percentages could be underestimated due to the fact that many older patients are excluded from clinical trials with intensive chemotherapy based on poor performance status. For this reason, outcome and clinical features at baseline in different clinical studies demonstrate a great variability. In this paper we review clinical results obtained in elderly APL patients with conventional chemotherapy and with more recent, alternative approaches.

## Results With Frontline Therapy with ATRA and Chemotherapy

Before the advent of ATRA, complete remission (CR) rates obtained in elderly APL with chemotherapy alone were in the range of 50%.^17^–^19^ Several trials reported the efficacy of all trans-retinoic acid (ATRA) followed by chemotherapy or concomitantly combined. The European APL group reported the results of the randomized APL 93 trial in which elderly patients (from 66 to 75 years) received ATRA followed by chemotherapy. CR in patients older than 60 years was high (86%) but significantly lower than that reported in younger patients (94%) and even in patients older than 70 years, the CR rate remained high (85%).^14^ The European APL group updated and reported separately the results of APL93 trial in elderly patients and confirmed the overall lower CR rate as compared to younger patients and a higher incidence of early death in this subset, although patients with higher WBC count (>10,000/μL) were not represented. The 4-year incidence of relapse was 15.6% in adults older than 60 and 23.2% in younger adults although most elderly patients received less intensive consolidation chemotherapy. However, 18.6% of the patients older than 60 years who entered remission died in CR, mainly from sepsis during consolidation course or maintenance treatment, as compared to 5.7% of younger adults. Therefore, 4-year OS was 57.8% in the elderly compared to 78% in younger patients.^20^ The GIMEMA group treated 134 elderly patients (median age 66 years) with ATRA and idarubicin, reporting a CR rate of 86% and a death rate of 12% during induction. After achievement of CR, 67 patients received three consolidation courses, whereas 39 patients entered in an amended protocol and received only the first cycle: of the first series, 18 patients were withdrawn due to toxicity and 9 patients died in CR, whereas in the second series two patients died in CR. The results suggested that less intensive chemotherapy in the setting of elderly APL allows significant reduction of treatment-related toxicity maintaining therapeutic efficacy.^21^ To reduce treatment-related toxicity in the elderly, the GIMEMA group started in 1997 an amended protocol for patients aged >60years, with the same induction (ATRA+idarubicin) as in younger patients, followed by a single consolidation course (idarubicin plus cytarabine) instead of 3, and by maintenance with intermittent ATRA. The trial enrolled 60 patients of whom 54 (90%) achieved haematological remission and six died during induction. Four additional patients died in CR from haemorrhage and infection prior or during consolidation therapy. Eleven patients relapsed at a median time of 17.5 months from CR. The 5-year OS, disease-free survival (DFS) and CIR rates were 68.5%, 64.6% and 27.4%, respectively. The results of the trial showed improved OS compared to original protocol (68.5% vs 56%) due to reduction of non-relapse mortality. CIR rate remained similar to that of previous trial in spite of reduced intensity of chemotherapy. The difference in maintenance therapy did not seem to influence the outcome between the original and the amended trial.^22^ The Spanish PETHEMA group reported in 2004 their experience in patients aged > 60 years treated with ATRA plus idarubicin for induction, 3 anthracycline-based courses for consolidation followed by maintenance including ATRA. CR was achieved in 84% of patients, but 7 patients died in remission during consolidation and maintenance treatment. The group reported 6-year cumulative incidence of relapse, leukemia-free survival, and disease-free survival rates of 8.5%, 91%, and 79%, respectively. The authors reported in this series a higher incidence of low-risk patients as compared to younger patients, which in part may explain the low relapse rate observed.^23^ The Japanese cooperative group JALSG compared the outcome of elderly patients with that of younger APL patients and confirmed a lower CR rate due to more early deaths. Forty-six patients (16.3%) out of 302 patients registered were described, median age 63 years. A comparison between younger and older patients did not reveal differences in terms of relapse risk at baseline (similar prevalence of high-risk) or other clinical features, except for lower platelet count, lower serum albumin and worse performance status in elderly patients. As reported by PETHEMA group 24, early deaths in the Japanese study were associated with low performance status at baseline and low albumin level. Cumulative incidence of relapse was similar between elderly and younger patients as reported also by the GIMEMA and the European APL group, but with 10-year OS was inferior (63% and 82% in older and younger patients, respectively).^25^ The European APL Group also reported that 10 year-OS in elderly patients was lower than that of the whole population (58.1% *vs* 77%) and the major cause of death in their elderly patients was sepsis during myelosuppression. The German AML Cooperative Group registered 91 elderly APL patients since 1994 and recently reported the outcome in this subset. Sixty-eight patients were treated in clinical trials and 23 were non-eligible for intensive chemotherapy. Fifty-six patients received ATRA associated to TAD (6-thioguanine, cytarabine and daunorubicine) as induction therapy followed by consolidation and maintenance. Fourteen patients received an intensification therapy with HAM schedule (high-dose cytarabine plus mitoxantrone). The early death rate was 48% in non-eligible patients and 29% for patients enrolled in clinical trials, but approximately 30% of patients had high-risk features at baseline. Seven-year OS, EFS and relapse-free survival (RFS) were 45%, 40% and 48% in patients treated with TAD schedule whereas beneficial effect in terms of RFS was reported (83%) for patients who received intensification.^26^

## Results with Arsenic Trioxide and Gemtuzumab Ozogamicin

Arsenic trioxide (ATO) combined or not with ATRA has proven highly effective in APL and is known to carry considerably less toxicity as compared to conventional chemotherapy-based approaches. Given that chemotherapy-related toxicity is a major issue in elderly APL, ATO seems an attractive option for frontline therapy in this setting. Considering the absence of myelosuppressive effects during post-remission therapy, ATO treatment can therefore not only shorten the duration of hospitalization but also avoid deaths from infection and serious infectious complications in CR, therefore prolonging the survival time in the elderly patient.^27^ The Chinese group reported the long-term outcome of 33 elderly patients (aged > 60 years, range 60–79, only 5 patients considered at high-risk at baseline) treated with ATO as single agent for up to 4 years. Eighty-eighth % of patients achieved CR and the most common adverse event was leukocytosis, developed in 64% of patients with serious differentiation syndrome being observed in only 5 cases. Twenty-eight patients proceeded to post-remission therapy: adverse effects during therapy were mild, and transient and none of the treated patients died from ATO-related toxicities. With a median follow-up of 99 months, the 10-year cumulative incidence of relapse, OS, DFS, and cause-specific survival were 10.3%, 69.3%, 64.8%, and 84.8%, respectively, which are comparable with those reported in the younger APL population.^28^

GO is a recombinant humanized immunoglobulin G4 (IgG4) anti-CD33 monoclonal antibody (hP67.6) conjugated to N-acetyl-gamma calicheamicin dimethyl hydrazide, a naturally potent antibiotic.^29^ Several reasons account for the high efficacy of GO-based treatment in APL: the disease is characterized by a consistent phenotypic profile, with negative staining for HLA-DR and CD34 and strong expression of CD33; calicheamicin is a potent drug, similar to anthracyclines, which are known to be highly effective in APL; lack of, or very low expression in APL blasts of the Pgp 170.^30^–^33^ In 2004, our group reported on the efficacy of GO as a single agent (at the dose of 6 mg/mq) in 16 APL patients who had relapsed at the molecular level: molecular remission was obtained in 9 out of 11 (91%) patients tested after 2 doses and in 13 of 13 patients (100%) tested after the third dose.^34^ Our group also reported a preliminary experience in 3 unfit elderly APL patients treated for molecular relapse with GO at low dose (3 mg/m^2^).^35^ The first patient was in third molecular relapse and was pre-treated with GO at 6 mg/m^2^ for first molecular relapse. He was retreated with 2 doses of GO and remained in complete remission for 10 months and finally died for reasons not correlated with the disease. The second patient, considered unfit for chemotherapy due to another concomitant neoplasia, received three courses of consolidation with GO and maintained a long-lasting molecular response. The third patient was considered not eligible for intensive chemotherapy due an antecedent cardiac ischemia: he received three cycles of GO obtaining complete molecular remission and remained in complete molecular remission after 14 months. Finizio et al reported the case of an elderly patient not eligible for intensive chemotherapy due to severe cardiac failure and chronic anticoagulant therapy. After an induction therapy with ATRA alone at standard dose for a prolonged time (80 days), the patient received GO at the dose of 6 mg/m^2^ monthly for two months as consolidation therapy and remained in molecular remission for 29 months.^36^ Unfortunately, GO was withdrawn in 2010 after the results of randomized study by SWOG showing no improvement in efficacy and increased toxicity in the setting of elderly AML.

## Treatment of Very Elderly Patients (> 70 years)

Disperati et al. reported 13 patients diagnosed between 1999 and 2006 with a median age of 78 years (range 71–87), treated with ATRA associated to chemotherapy (cytarabine plus daunorubicine). Ninety-two % of patients entered CR and received consolidation with two courses of chemotherapy and maintenance with ATRA for 9 months. The reported 2-year OS was 76% with 10% death rate in the post-remission phase.^37^ Our group reported 12 patients aged > 70 years (median age 74.7 years) followed in a single center between 1991 and 2008. According to the Sanz s relapse risk score, 7 patients were classified as low and 5 patients as intermediate risk. Eight patients received standard induction with ATRA and idarubicin whereas 4 patients received only ATRA, but during induction they needed the association of chemotherapy for severe leukocytosis. All patients achieved hematologic and molecular remission and differentiation syndrome occurred in 2 patients. All but one patient received consolidation (chemotherapy alone in 7 patients, chemotherapy plus ATRA in 3 patients and ATRA alone in 1 patient).^38^ Four patients experienced disease relapse. Ferrara and colleagues reported 34 unselected elderly patients aged over 60 years (median age 70 years) treated for induction with ATRA plus chemotherapy or with ATRA alone. Of these, 23 (68%) fulfilled inclusion criteria of the AIDA protocol, whereas 11 (32%) received a personalized treatment. CR was reached in 68% of patients that were consolidated with the first cycle of the AIDA schedule (idarubicin plus cytarabine) or with gentuzumab ozogamicin. Median OS reported was 38 months and none of the patients died during post-remission phase.^39^

## Relapsed Patients

ATRA combined with chemotherapy can yield second molecular CR in a large proportion of older relapsed APL patients, but this approach is associated with significant toxicity and profound myelosuppression.^40^ Although only few experiences have been reported in the subset of elderly patients who relapse, ATO is reported to yield at least equivalent hematological and molecular results with limited toxicity. Soignet et al reported in 2001 the US multicenter experience and 8 out of 40 patients were aged over 60 years. Six of them (75%) achieved a CR as compared to 26/32 younger (81%) patients. OS in elderly patients was 38% at 18 months compared to 66% in the overall population, probably due to the fact that older patients did not receive transplant procedures after CR.^41^ Recently we reported on the efficacy of prolonged therapy with combined ATO and ATRA in relapsed patients: 3 out of 9 patients were aged over 60 years and received 5 cycles of ATO and ATRA as reported by Estey et al 42. Only 1 patient experienced electrolyte abnormalities during ATO administration, but none of the patients had other toxicities and all maintained prolonged molecular CR.^43^

## Conclusions

APL is relatively rare in elderly patients and ATRA combined with chemotherapy has considerably improved survival also in this setting. Compared to younger patients, however, outcome in elderly APL is inferior due to higher incidence of early death and of death in CR. Distinct to other AML subsets, relapses are quite uncommon in elderly APL patients, and the disease is curable in the majority of cases. However, it is likely that some high risk patient are excluded from entering clinical trials. For the frail patients who are considered unfit for conventional treatment, ATO with or without ATRA might be a reasonable alternative to the standard ATRA plus chemotherapy approach, although supporting scientific data currently are limited, particularly with respect to rates of remission and complications such as APL differentiation syndrome. It is foreseen that the ATO plus ATRA combination which very recently showed similar efficacy as compared to ATRA and chemotherapy in younger patients, will be explored in the near future in larger studies involving elderly patients.

## Figures and Tables

**Table 1:** Outcome results in main studies on elderly APL

Author	Year	N°Pts	Relapse risk	Median age (yrs)	Induction	Consolidation	CR (%)	OS (%)	DFS (%)
Mandelli	2003	86	Low nr Int nr High 16%	65.8	ATRA + Ida	3 cycles	86	56 (6 yrs)	59 (6 yrs)
Sanz	2004	104	Low 37% Int 43% High 20%	68.0	ATRA + Ida	3 cycles	84	NR	79 (6 yrs)
Ades	2005	129	Low nr Int nr High 0%	66.0	ATRA + Dauno+ AraC	2 cycles	86	57.8 (4 yrs)	53 (4 yrs)
Latagliata	2011	60	Low 34.6% Int 51.9% High 13.5%	66.1	ATRA + Ida	1 cycle	90	68.5 (5 yrs)	64.6 (5 yrs)
Ono	2012	46	Low 28% Int 52% High 20%	63.0	ATRA + Ida- AraC	3 cycles	89	63 (10 yrs)	65 (10 yrs)
Lengfelder	2013	56	Low 27% Int 42% High 31%	67.0	ATRA + TAD	1 cycle	82	45 (7 yrs)	48% (7 yrs)

Legend: ATRA, All-trans retinoic acid. Ida, idarubicin. AraC, cytosine arabinoside. TAD, thioguanine, cytocine arabinoside, daunorubicin. CR, complete remission. OS, overall survival. DFS, disease-free survival. Nr= not reported
